# Isolation and enzyme bioprospection of endophytic bacteria associated with plants of Brazilian mangrove ecosystem

**DOI:** 10.1186/2193-1801-3-382

**Published:** 2014-07-28

**Authors:** Renata A Castro, Maria Carolina Quecine, Paulo T Lacava, Bruna D Batista, Danice M Luvizotto, Joelma Marcon, Anderson Ferreira, Itamar S Melo, João L Azevedo

**Affiliations:** Center for Nuclear Energy in Agriculture (CENA), University of São Paulo, Piracicaba, SP Brazil; Department of Genetics, Escola Superior de Agricultura, “Luiz de Queiroz” (ESALQ), University of São Paulo, Piracicaba, SP Brazil; Department of Morphology and Pathology, Center for Biological and Health Sciences, Federal University of São Carlos (UFSCar), São Carlos, SP Brazil; Brazilian Agricultural Research Corporation – Embrapa Agrosilvopastoral, Sinop, MS Brazil; Laboratory of Environmental Microbiology, CNPMA — Embrapa Environment, Jaguariúna, SP Brazil

**Keywords:** Mangrove, Endophytes, Bacteria, Biotechnological potential, Enzymes

## Abstract

The mangrove ecosystem is a coastal tropical biome located in the transition zone between land and sea that is characterized by periodic flooding, which confers unique and specific environmental conditions on this biome. In these ecosystems, the vegetation is dominated by a particular group of plant species that provide a unique environment harboring diverse groups of microorganisms, including the endophytic microorganisms that are the focus of this study. Because of their intimate association with plants, endophytic microorganisms could be explored for biotechnologically significant products, such as enzymes, proteins, antibiotics and others. Here, we isolated endophytic microorganisms from two mangrove species, *Rhizophora mangle* and *Avicennia nitida*, that are found in streams in two mangrove systems in Bertioga and Cananéia, Brazil. *Bacillus* was the most frequently isolated genus, comprising 42% of the species isolated from Cananéia and 28% of the species from Bertioga. However, other common endophytic genera such as *Pantoea, Curtobacterium* and *Enterobacter* were also found. After identifying the isolates, the bacterial communities were evaluated for enzyme production. Protease activity was observed in 75% of the isolates, while endoglucanase activity occurred in 62% of the isolates. *Bacillus* showed the highest activity rates for amylase and esterase and endoglucanase. To our knowledge, this is the first reported diversity analysis performed on endophytic bacteria obtained from the branches of mangrove trees and the first overview of the specific enzymes produced by different bacterial genera. This work contributes to our knowledge of the microorganisms and enzymes present in mangrove ecosystems.

## Background

Mangroves are a tropical coastal biome that is located in the transition zone between the land and the sea where the vegetation is dominated by a particular group of plant species (Zhou et al. 2006). This ecosystem is characterized by periodic tidal flooding, making environmental factors such as salinity and nutrient availability highly variable and resulting in unique and specific environmental characteristics (Holguin et al. 2006). Thus, the mangrove ecosystem provides a distinct environment harboring diverse groups of microorganisms (Thatoi et al. 2013; Sivaramakrishnan et al. 2006). Although the mangrove ecosystem is rich in microbial diversity, less than 5% of the species present have been described; in many cases, neither their ecological role nor their technological potential is known (Thatoi et al. 2013).

Various groups of bacteria are typically present in the mangrove ecosystem (Holguin et al. 2001) where they perform diverse activities including photosynthesis, nitrogen fixation, and methanogenesis (Das et al. 2006). Bacterial communities can be found living freely in mangrove sediments (Roy et al. 2002; Dias et al. 2009a, 2010) or as endophytes associated with the native flora (Garcias-Bonet et al. 2012; Janarthine et al. 2010; Liu et al. 2010; Feng et al. 2009).

Endophytes are microorganisms that live inside of plants without causing any harm to their hosts (Azevedo and Araújo 2007). Endophytic bacteria have been isolated from root nodules and the stems, leaves and fruits of a wide variety of plant species including citrus (Araujo et al. 2001), sugarcane (Gangwar and Kaur 2009), maize (Araujo et al. 2000), eucalyptus (Procopio 2009; Ferreira et al. 2008), soybean (Kuklinsky-Sobral et al. 2005; Assumpção et al. 2009), and strawberry (Dias et al. 2009a), among others. However, some endophytic communities remain unexplored in studies describing the bacterial communities from tropical native plants. Consequently, studies on the endophytic bacteria of plants from different ecosystems (mangroves, for example) offer a great opportunity to discover new compounds and resources with biotechnological potential that can be exploited (Sivaramakrishnan et al. 2006). Microorganisms from mangrove ecosystems contain useful enzymes, proteins, antibiotics and salt tolerant genes, all of which have biotechnological significance (Thatoi et al. 2013).

Microorganisms are important to enzymatic production processes because of their high production capability, low cost and susceptibility to genetic manipulation. There is strong biotechnological interest in microbial enzymes in several fields including food processing, detergent and textile manufacturing, agricultural and pharmaceutical research, medical therapy and molecular biology (Stamford et al. 1998; Carrim et al. 2006; Quecine et al. 2008 and 2011; Ferreira-Filho et al. 2012). Little is known about bacterial mangrove communities, but these microorganisms may have high biotechnological potential. Consequently, for the first time, the present work isolated and identified the endophytic bacteria taken from the branches of the two major species of mangrove plants, *Rhizophora mangle* (red mangrove) and *Avicennia* nitida (white mangrove), that are found in two different mangrove areas of São Paulo State, Brazil (Cananéia and Bertioga). Furthermore, this study investigated the potential of the bacterial isolates to produce enzymes with industrial interest, such as amylase, esterase, lipase, protease and endoglucanase.

## Results

### Bacterial isolation and molecular identification

Following isolation, bacterial abundance was estimated based on plant species and sampled area. The bacterial densities were very similar in all samples: approximately 10^5^ CFU g tissue^-1^ for both mangrove species analyzed (*R. mangle* and *A. nitida*) and both sites (Bertioga and Cananéia).

The bacterial communities clearly differed in composition according to the mangrove species and locality. Bacterial isolates were classified into 14 genera. The isolates from Cananéia were grouped into 8 genera with a predominance of *Bacillus* sp. (42.1%) followed by *Enterobacter* (10.5%), *Chryseobacterium* (10.5%), *Xanthomonas* (10.5%), and others. The isolates from Bertioga were grouped into 9 genera with a predominance of *Bacillus* (28.6%) followed by *Curtobacterium* (14.3%), *Alcaligenes* (14.3%), *Ochrobactrum* (9.5%), *Novosphingobium* (9.5%), and others. Interestingly, *Ochrobactrum* and *Microbacterium* were only found in *R. mangle* branches, while *Novosphingobium* was isolated only from *A. nitida* branches (Table 1). Major isolates belongs to Bacilli class (35%), followed by Gamaproteobacteria class (25%). All isolates was classified at genus level, to avoid an incorrect classifications at specie level (Table 2).

**Table 1:** **Distribution of the genera of endophytic bacteria isolated from Brazilian mangrove forests**

***Genera***	Sample ^a^			
	Cananéia		Bertioga	
	***A. nitida***	***R. mangle***	***A. nitida***	***R. mangle***
*Bacillus*	5	3	4	2
*Enterobacter*	-	2	-	-
*Pantoea*	-	1	-	-
*Brevundimonas*	1	-	-	-
*Microbacterium*	1	-	1	-
*Chryseobacterium*	3	-	-	-
*Novosphingobium*	1	-	1	1
*Xanthomonas*	2	-	-	-
*Erwinia*	-	-	1	-
*Alcaligenes*	-	-	3	-
*Ochrobactrum*	-	-	-	3
*Curtobacterium*	-	-	-	3
*Stenotrophomonas*	-	-	-	1
*Sphingopyxis*	-	-	1	-
**Diversity parameters** ^**b**^				
S	6	3	6	5
N	13	6	11	10
D	2.35	1.12	2.08	2.17
H'(loge)	1.74	1.00	1.59	1.69
1-Lambda'	0.84	0.72	0.84	0.89

^a^Number of isolates from each plant and sample location.

^b^Diversity parameters: S, total taxons; N, total individuals; D, species richness (Margalef); H' (loge), Shannon; and 1-λ, Simpson diversity indices for endophytic bacterial communities isolated from *A. nitida a*nd *R. mangle* branches sampled in Cananéia and Bertioga, Brazil.

**Table 2:** **Identification by partial sequencing of 16S rDNA of isolates from mangrove sediments**

Isolates	Class	Genus	Coverage	Identidade	Nearst Taxa
MBR2.20	Actinobacteria	*Curtobacterium*	100	100	*Curtobacterium oceanosedimentum*
MBR2.21			100	100	*Curtobacterium oceanosedimentum*
MBR2.22			100	99	*Curtobacterium flaccumfaciens*
MCA2.54		*Microbacterium*	100	100	*Microbacterium arborescens*
MBA2.52			100	99	*Microbacterium oleivorans*
MCR2.49	Bacilli	*Bacillus*	100	99	*Bacilus megaterium*
MCR2.51			100	100	*Bacillus altitudinis*
MCR2.56			100	94	*Bacillus vallismortis*
MCA2.42			100	99	*Bacillus safensis*
MCA2.51			100	100	*Bacillus pumilus*
MCA2.56			100	100	*Bacillus subtilis*
MCA2.53			100	100	*Bacillus subtilis*
MCA2.41			100	97	*Bacillus safensis*
MBR2.4			100	98	*Bacillus amyloliquefaciens*
MBR2.41			100	95	*Bacillus safensis*
MBA2.9			94	96	*Bacillus pumilus*
MBA2.33			100	100	*Bacillus safensis*
MBA2.4			100	99	*Bacillus pumilus*
MBA2.18			100	99	*Bacillus pumilus*
MCA2.12	Alphaproteobacteria	*Novosphingobium*	100	99	*Novosphingobium* sp.
MBR2.7			100	99	*Novosphingobium resinovorum*
MBA2.41			100	99	*Novosphingobium* sp.
MCA2.9		*Brevundimonas*	100	99	*Brevundimonas vesiculares*
MBR2.46		*Ochrobactrum*	100	100	*Ochrobactrum pseudogrignonense*
MBR2.39			100	100	*Ochrobactrum pseudogrignonense*
MBR2.33			100	99	*Ochrobactrum pseudogrignonense*
MBA2.44		*Sphingopyxis*	100	100	*Sphingopyxis* sp.
MBA2.27	Betaproteobacteria	Alcaligenes	100	100	*Alcaligenes faecalis*
MBA2.16			100	99	*Alcaligenes faecalis*
MBA2.15			100	99	*Alcaligenes faecalis*
MCR2.37	Gammaproteobacteria	*Enterobacter*	100	98	*Enterobacter hormaechei*
MCR2.29			100	99	*Enterobacter* sp.
MCA2.21		*Chryseobacterium*	100	99	*Chryseobacterium* sp.
MCA2.27			100	99	*Chryseobacterium* sp.
MCA2.23			100	100	*Chryseobacterium* sp.
MCA2.20		*Xanthomonas*	100	100	*Xanthomonas campestris*
MCA2.39			100	99	*Xanthomonas* sp.
MCR2.33		*Pantoea*	100	100	*Pantoea dispersa*
MBR2.29		*Stenotrophomonas*	100	99	*Stenotrophomonas maltophilia*
MBA2.19		*Erwinia*	100	98	*Erwinia tasmaniensis*

The diversity parameters for each sample were very similar, as the comparative analysis of the Shannon diversity indices for *A. nitida* versus *R. mangle* and Bertioga versus Cananéia did not differ statistically according to t-test analysis (p *<* 0.05) (Table 3).

**Figure 1:**
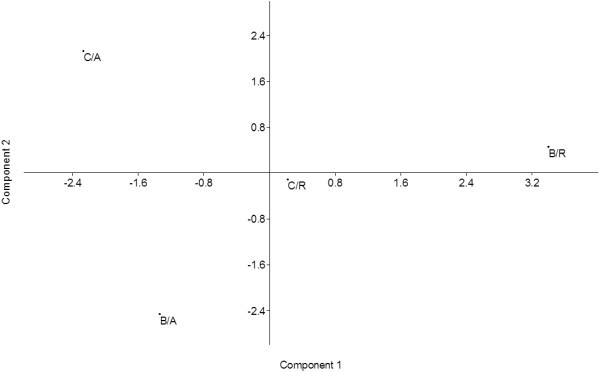
**Principal component analysis (PCA) based on the molecular identification of bacterial isolates from Cananéia–** ***Avicennia nitida*** **(C/A), Cananéia–** ***Rhizophora mangle*** **(C/R), Bertioga–** ***Avicennia nitida*** **(B/A), and Bertioga–** ***Rhizophora mangle*** **(B/R).** The values on the axes indicate the variance explained.

Despite the similar values for the Shannon diversity indices, principal component analysis clearly demonstrated that the bacterial communities are specific according to locality and plant sample (Figure 1).

**Table 3:** **Diversity t-test (** ***p*** **< 0.05) for bacterial communities isolated from** ***A. nitida*** **and** ***R. mangle*** **branches**

	Bertioga VS Cananeia			
	Shannon index	Variance	***P***	T
**Whole sample**			0.644	0.464 (ns)
***Avicenia***	1.79	0.037		
***Rizhophora***	1.67	0.037		
	***Avicenia vs Rizopphora***			
**Whole sample**			0.432	0.803 (ns)
**Bertioga**	1.87	0.031		
**Cananeia**	1.64	0.051		

ns - non-significant.

The mangroves are located in Bertioga and Cananéia, SP, Brazil.

### Bacterial enzymatic activity

The Enzymatic Index (EI) for all isolates was obtained following observation of their ability to produce at least 1 of 5 evaluated enzymes. Amylolytic activity was observed in 45%, esterasic activity in 17.5%, lipolytic activity in 52.5%, proteolytic activity in 75% and endocellulolytic activity in 62.5% of the tested isolates (Table 4).

**Table 4:** **Enzymatic activity of the endophytic bacteria isolated from mangrove forests**

Genus	Isolates ^a^	Enzymatic Index (EI) ^b^				
		Amylase	Esterase	Lipase	Protease	Endoglucanase
*Alcaligenes*	MBA2.15	-	-	2.23 f	1.43 e	-
	MBA2.27	-	-	4.25 c	-	1.65 c
	(MBA2.16)	-	-	1.73 g	2.15 c	1.98 b
*Bacillus*	MCA2.41	-	-	1.35 g	-	1.60 c
	MCA2.42	-	-	2.80 e	2.48 b	2.58 a
	MBA2.18	-	-	4.38 c	1.23 f	2.08 b
	MBA2.33	2.50 b	-	4.80 c	-	2.73 a
	MBA2.4	-	-	2.55 f	1.75 d	2.80 a
	MBA2.9	1.58 e	-	-	-	-
	MBR2.4	1.55 e	-	-	-	2.80 a
	MBR2.41	2.25 c	1.60 b	-	1.33 f	1.60 c
	MCA2.51	-	-	3.13 d	1.58 d	2.75 a
	MCA2.53	2.75 a	1.63 b	-	1.10 f	2.00 b
	MCA2.56	2.28 b	-	1.63 g	1.23 f	1.90 b
	MCR2.49	2.70 a	1.58 b	-	1.65 d	-
	MCR2.51	-	-	3.13 d	1.58 d	2.75 a
	MCR2.56	2.28 b	1.85 a	-	1.23 f	2.23 b
*Brevundimonas*	MCA2.9	1.80 d	-	-	2.18 c	-
*Chryseobacterium*	MCA2.21	2.43 b	-	-	1.58 d	-
	MCA2.23	-	-	2.30 f	-	-
	MCA2.27	2.53 b	-	-	1.53 e	-
*Curtobacterium*	MBR2.20	-	-	-	2.75 a	-
	MBR2.21	-	-	-	1.35 e	2.38 a
	MBR2.22)	-	1.60 b	-	1.05 g	1.43 c
*Enterobacter*	MCR2.29)	-	-	-	1.78 d	1.73 c
	MCR2.37	-	-	2.50 f	1.35 e	-
*Erwinia*	MBA2.19	1.63 e	1.30 c	6.83 a	-	-
*Microbacterium*	MBA2.52	1.73 d	-	-	1.15 f	2.13 b
	MCA2.54	-	-	1.48 g	1.75 d	2.93 a
*Novosphingobium*	MBA2.41	1.80 d	-	5.13 b	-	2.13 b
	MBR2.7	2.50 b	-	3.48 d	1.88 d	-
	MCA2.12	2.78 a	-	-	1.65 d	-
*Ochrobactrum*	MBR2.33	-	-	-	2.3 c	2.28 b
	MBR2.39	-	-	3.08 d	-	2.60 a
	MBR2.46	-	-	2.90 e	-	2.90 a
*Pantoea*	MCR2.33	-	-	-	1.68 d	2.23 b
*Sphingopyxis*	MBA2.44	-	-	4.75 b	2.53 b	-
*Stenotrophomonas*	MBR2.29	1.43 f	-	2.50 e	1.55 d	2.63 a
*Xanthomonas*	MCA2.20	-	1.38 c	-	2.5 b	-
	MCA2.39	1.15 g	-	-	1.7 d	-

^a^Bacterial identification at the genus level with the name of the isolates in parentheses. The isolates were named according to host and the location of the samples: MCR, Mangrove Cananéia *Rhizophora mangle*; MCA, Mangrove Cananéia *Avicennia nitida*; MBR, Mangrove Bertioga *Rhizophora mangle*; MBA, Mangrove Bertioga *Avicennia nitida.*

^b^The enzymatic index was measured by determining the ratio of degradation halo diameter/bacterial colony diameter. Values with the same letter within a column are not significantly (p < 0.05) different according to the Scott-Knott test. The results represent the means of four replicates for each isolate.

Some isolates exhibited high enzymatic performance: *Bacillus* (MCR2.56) showed one of the highest amylase and esterasic activities with EI of 2.28 and 1.85, respectively. Six *Bacillus* isolates (MCR2.51, MCA2.42, MCA2.51, MBR2.4, MBA2.33, and MBA2.4) showed high endocellulolytic activities. The following genera also demonstrated high enzymatic productivity: *Curtobacterium* (MBR2.21), *Ochrobactrum* (MBR2.46), *Microbacterium* (MCA2.54), *Brucella* (MBR2.39), and *Stenotrophomonas* (MBR2.29) showed high endoglucanase activity; *Erwinia* sp. (MBA2.19) showed lipase activity; and *Curtobacterium* sp. (MBR2.20) showed protease and other activities.

## Discussion

Currently, marine microorganisms are attracting increasing attention as a resource for new enzymes because the enzymes derived from marine microbes are relatively more stable and active than the corresponding enzymes derived from plants or animals (Lam 2006; Bull et al. 2000). Chen et al. (2012) isolated endophytic bacteria from 4 species of aquatic plants: *Phragmites communis*, *Potamogeton crispus*, *Nymphaea tetragona* and *Najas marina.* The isolated bacteria were classified into 12 genera in the *Gammaproteobacteria*, *Bacilli*, *Alphaproteobacteria*, *Flavobacteria* and *Actinobacteria.*However, studies focusing on the isolation and characterization of endophytic bacteria from native plant specimens from the Brazilian mangrove biome are rare. To our knowledge, the present work is the first report on the isolation, identification and enzymatic characterization of endophytic microorganisms from *R. mangle* and *A. nitida* in Brazil.

*Bacillus* was the most abundant genus isolated from all samples as described previously (Liu et al. Liu et al. 2010). Our data corroborate the results obtained by Ando et al. (2001) who isolated a large number of *Bacillus* sp. from mangrove sediments in Japan and reported the possible ability of these isolates to degrade organic pollutant compounds by fermentation. Various *Bacillus* sp. have also been isolated from several fish, mollusks, sediments and marine waters in Canada (Schulze et al. 2006). Ravikumar et al. (2010) isolated many endophytic bacteria from mangrove halophytic plants collected from the Pichavaram mangrove forest in India. Among the isolates, the authors identified two endophytes, *B. thuringiensis* (MB4) and *B. pumilus* (MB8), which were able to control many bacterial and fungal pathogens. Similarly, the endophytic strain *B. amyloliquefaciens* (RS261) is a biological agent isolated from the leaf of *R. stylosa* (Feng et al. 2009).

We found additional genera, such as *Curtobacterium* and *Xanthomonas*, colonizing specific plant species from specific localities. Interestingly, *Microbacterium* was isolated in both Bertioga and Cananéia, but only from *A. nitida*, suggesting host-specificity. Free-living *Microbacterium* has been isolated from the same area from mangrove sediments (Dias et al. 2009a).

The diversity analysis clearly demonstrated that the evaluated parameters from each sample were similar; however, the bacterial communities are totally dependent on the host and the sampled site. Dias et al. (2010) assessed the bacterial diversity of mangrove sediments from São Paulo State by culture and also independent-culture methods. The authors observed that the location within the mangrove was determinant for all of the fractions of the community studied, which corroborates our data.

In further another point that should be explored is that mangrove microorganisms demonstrate a diverse range of enzymatic activities and are capable of catalyzing various biochemical reactions using novel enzymes (Thatoi et al. 2013; Dias et al. 2009b). Halophilic microorganisms in particular possess many hydrolytic enzymes and are capable of functioning under conditions that lead to precipitation or denaturation of most proteins (Ventosa and Nieto 1995).

As applied here, the enzymatic index was a fast and practical tool for selecting and comparing the enzyme production of different bacterial isolates. Values above 1.0 were indicative of enzyme secretion (Carrim et al. 2006). In the present study, all of the isolates showed the ability to produce at least one of five evaluated enzymes, proving their potential for industrial applications.

Dias et al. (2009b) evaluated the bacterial community from mangrove sediments in Brazil and found a predominance of organisms from the orders *Vibrionales* and *Bacillales*. These isolates were also able to produce diverse extracellular enzymes such as amylases, proteases, esterases and lipases, as found in the present work as well. The *Bacillus* spp. strains from mangrove sediments reached 5.53 and 5.06 amylase and protease enzymatic indexes, our *Bacillus* spp. strains reached 2.75 enzymatic indexes to both enzymes. However, the *Bacillus* sp*.* (MBA2.33) shows a lipase enzymatic index of 4.8, higher than of the best lipase producer *Bacillus* sp. 1A339 strain (lipase enzymatic index 3.88) from mangrove sediment (Dias et al. 2009b)

In a recent study, Khianngam et al. (2013) isolated and screened endophytic bacteria from mangrove plants in Thailand for the presence of hydrolytic enzymes. Twenty isolates showed activities associated with proteases, lipases, amylases or cellulases. The Rhf-2 strain, which was isolated from the fruit of *Rhizophora mucronata*, produced all of these enzymes; the strain was later identified as *Bacillus safensis*.

More than 50% of the isolates evaluated in the present work produced endoglucanase. Cellulases are commercially produced by several industries globally and are widely used in food, animal feed, fermentation, agriculture, pulp and paper, and textile applications (Kuhad et al. 2011).

Tabao and Moasalud (2010) evaluated the bioprospecting potential of the bacterial community found in mangroves in the Philippines, and the following four promising cellulase producing bacteria were identified: *B. cereus*, *B. licheniformis*, *B. pumilus* and *Bacillus* sp. Our study also identified many *Bacillus* sp. showing strong enzymatic production. Other isolated genera further demonstrated high productivity for proteases (*Curtobacterium* MBR2.20) and lipases (*Erwinia* sp. MBA2.19), suggesting their application as detergent additives (Jurado et al. 2007; Saeki et al. 2007).

## Conclusions

Our work has improved the few information about the diversity of the endophytic bacterial communities from mangrove trees, suggesting a specific interaction between endophytes and mangrove host plants. Moreover, mangrove microorganisms demonstrated a diverse range of enzymatic activities that have been poorly explored biotechnologically. The isolates probably are capable of catalyzing various biochemical reactions using probable novel enzymes that should be further investigated in the future.

## Methods

### Sample collection

Branches from five randomly selected *Rhizophora mangle* and five *Avicennia nitida* trees located in Bertioga (S 23° 54′ 01.1″/WO 46° 15′ 01.3″) and also Cananéia (S 25° 05′ 87″/WO 47° 57′ 70″), São Paulo State, Brazil, were sampled for bacterial isolation. The samples were collected stored in plastic bags and taken to the laboratory for bacterial isolation.

### Bacterial isolation

The tree samples were washed with water to remove adherent particles and were superficially disinfected according to Araújo et al. (2002). Then, the samples were cut into fragments, and roughly 1 g was triturated in the presence of 5 mL of PBS (Phosphate Buffered Saline) buffer, transferred to a 15 mL tube and shaken for 1 hour at 180 rpm. After obtaining the suspension of microorganisms, dilutions were made in PBS buffer, and aliquots of 100 μL were inoculated onto 5% Tryptic Soy Agar (TSA, Difco) medium supplemented with Benomyl (50 μg mL^-1^) to inhibit fungal growth. The plates were incubated at 28°C for 1–7 days until growth was observed, upon which the number of colony-forming units (CFU) were counted and the population density estimated. Aliquots of 1.0 mL of the last wash water were also inoculated onto TSA medium to evaluate the effectiveness of the disinfection process, and only culture samples showing no growth were used.

Endophytic isolates were purified and inoculated into liquid 5% Tryptic Soy Broth (TSB, Merck) medium supplemented with glycerol (15% final concentration) and stored at -80°C for future experiments.

### Molecular identification of bacterial isolates

Bacterial isolates were identified by partial sequencing of 16S rDNA. The 16S rDNA was amplified directly from bacterial colonies grown on 10% TSA medium using the primers R1387 (5′-CGGTGTGTACAAGGCCCGGGAACG-3′) and PO27F (5′-GAGAGTTTGATCCTGGCTCAG-3′) (Heuer et al. 1997). The PCR reactions were performed in a 50 μL volume containing 31.8 μl deionized water, 5.0 μl buffer, 7.5 μl MgCl_2_, 4.0 μl dNTPs, 0.1 μl of each primer*,* 0.5 U Taq DNA polymerase and 1 μl template DNA. The reaction conditions for the thermocycler (PTC 200, MJ Research) were as follows: initial denaturation for 4 min at 94°C; followed by 35 cycles of 94°C for 30 s, annealing for 1 min at 62.5°C and primer extension for 1 min at 72°C; ending with a final extension for 7 min at 72°C. The reaction products were analyzed on an agarose gel (1.2%) together with a 1 Kb DNA molecular weight marker (Fermentas); the results were subsequently photographed.

The 16S rDNA PCR products (approximately 1400 bp in size) were purified using the polyethylene glycol method of Lis (1980) and sequenced at the ‘Instituto do Genoma Humano’ (USP, São Paulo, Brazil). Sequencing was performed using the primer 1387R (Heuer et al. 1997).

The bacterial identification was performed comparing the sequences deposited in the following public banks: Ribosomal Database Project II (RDP) (http://www.rdp.cme.msu.edu) using the Classifier program with 95% confidence (Wang et al. 2007) and GenBank from National Canter for Biotechnology Information-NCBI (http://www.ncbi.nlm.nih.gov) using, for this purpose, the BLASTn tool (Altschul et al. 1990). All of the bacterial sequences presented in this study were submitted to GenBank (accession numbers KF356428- KF356467).

### Enzymatic activity

To forty molecular identified bacteria the production and the semi-quantitative measurement activity of the following enzymes was analyzed: amylase, esterase, lipase, cellulase and protease. To determine enzymatic activities, isolates were initially grown in 5% TSA for 24 h at 28°C, after which 15 μl aliquots (OD_550nm_ = 0.05, approximately 10^8^ CFU/mL) dropped on specific media and incubated at 28°C. The Enzymatic Index (EI) was measured after 72 h of incubation and was expressed as the ratio between the halo diameter and the bacterial colony diameter (Hankin et al. 1971; Hankin and Anagnostakis 1975).

The amylolytic activity was measured according to Hankin and Anagnostakis (1975). The isolates were inoculated onto 5% TSA medium containing 1% soluble starch. After bacterial growth, 5 mL of a 1% iodine solution was added to each plate, allowing the visualization of clear halos around the colonies.

The medium described by Sierra (1957) was used to evaluate the lipolytic activity. To sterilized culture medium, previously sterilized Tween 80 or Tween 20 was added to a final concentration of 1% (v/v) to evaluate respectively the bacterial esterasic and lipase activity. The presence of halos was considered indicative of enzymatic activity.

To determine proteolytic activity, we used a culture medium containing skim milk according to Quecine (2010). The formation of a halo around the colony was considered indicative of proteolytic activity.

Finally, the cellulolytic activity of the isolates was evaluated according to Teather and Wood (1982). The isolates were grown on M9 medium (Sigma) containing 0.5% yeast extract and 1% carboxymethylcellulose (CMC). After bacterial growth, 10 mL of Congo red dye (1%) was added and the plates were washed with NaCl (5 M). The presence of a colorless halo around the colony was indicative of enzymatic activity.

### Data analysis

The matrices generated from bacterial identification at the genus level were used in the molecular analysis described below. “Primer 6” (Plymouth Marine, Primer, United Kingdom) was used to obtain the different diversity indices: S, total profile; N, profile value (band intensities); d, species richness (Margalef); H′ (loge), Shannon; and 1-λ, Simpson diversity.

Additionally, the t-test for the Shannon diversity index was realized using the PAST program (Hammer et al. 2001). Spatial distribution as determined by principal component analysis for the endophytic bacterial communities from *A. nitida* and *R. mangle* sampled from Bertioga and Cananéia was also determined using the PAST program. The axis values showed the percentage variance of each community.

The statistical analysis of enzymatic production was performed using the R program. A completely random design was used for all of the enzymatic assays. The EI values were measured using the ratio of halo diameter/bacterial diameter. All data were analyzed for significance (p < 0.05) using the Scott-Knott test.
